# Strategies for Teachers to Promote Physical Activity in Early Childhood Education Settings—A Scoping Review

**DOI:** 10.3390/ijerph18030867

**Published:** 2021-01-20

**Authors:** Toby C. T. Mak, Derwin K. C. Chan, Catherine M. Capio

**Affiliations:** 1 Faculty of Education and Human Development, The Education University of Hong Kong, Hong Kong, China; tctmak@eduhk.hk (T.C.T.M.); derwin@eduhk.hk (D.K.C.C.); 2 School of Public Health, The University of Hong Kong, Hong Kong, China; 3 Health Science Department, Ateneo de Manila University, Quezon City 1108, Philippines

**Keywords:** children, teacher, physical activity, early childhood education and care

## Abstract

Recent evidence has emphasized the importance of the early childhood years for developing lifelong physical activity patterns. As such, evidence-informed programs that create opportunities for young children to engage in physical activity are needed and education settings present an important context. This review aimed to identify strategies that are implemented by teachers to promote physical activity in early childhood education and care settings. This is a scoping review that followed the framework proposed by the Joanna Briggs Institute. Searches were conducted using the databases of PubMed, SCOPUS, PsycINFO, SPORT Discus, ERIC and Web of Science for publications up to September 2020. From a total of 8974 articles, 19 were deemed eligible. Ten types of strategies, performed by teachers with the intention to improve physical activity-related primary outcomes, were identified. Physical activity promotion by teachers in early childhood settings is recommended to take a multi-strategy approach, in conjunction with professional development training opportunities and continuous follow-up support for teachers. Future work is warranted to fill the evidence gap in other regions (e.g., Asia, Africa and South America) and strengthen the evidence base to establish best practice standards.

## 1. Introduction

Clear evidence shows that engaging in regular physical activity at a young age results in numerous benefits, such as associated improvements in physical, psychosocial and cognitive development domains and eventual academic performance (for example, see [1,2]). From a health perspective, adequate physical activity can not only reduce the risk of obesity, but it can also promote bone health and facilitate psychological, social and fundamental motor skill development for preschool-aged children [3,4,5]. Recent recommendations from the World Health Organization [6] suggest that young children should engage in a minimum of 180 min of physical activity and at most 60 min of screen-based sedentary time per day. Insufficient physical activity in children is likely to contribute to risks for chronic, non-communicable diseases that include osteoporosis, cancer and cardiovascular disease in later life [7,8]. Health behavior patterns established in early childhood are likely to track to adulthood and relative physical inactivity of children is likely to persist as they grow older [9,10]. As such, efforts in creating opportunities for young children to start engaging in physical activity at an early stage are warranted and evidence-informed programs are needed. In this paper, we would like to offer a scoping review on strategies or programs that teachers implement and deliver in school- or early childhood education and care (ECEC) center-based settings to promote physical activity in young children.

### 1.1. Background

The early childhood years comprise a vital period for developing individual physical activity patterns and experts recommend that children should take part in substantial amounts of physical activity through both structured and unstructured play [11]. Early childhood education settings are important venues for promoting physical activity in young children through opportunities such as active outdoor or free play [12,13,14] and educational strategies that raise interest in physical activity [15]. Early childhood educators who are adequately trained and enabled with knowledge are ideally placed to promote physical activity engagement amongst their pupils [16]. Their critical role is in providing quality opportunities and creating active experiences for children’s development within the school setting [17].

Programs that are implemented or facilitated by early childhood educators (instead of external specialists) are especially important because they are not dependent on external personnel or constraints that are associated with additional costs. As such, evidence-based recommendations for physical activity promotion implemented by teachers in early childhood education contexts are important. Other strategies in education settings include the adaptation of the physical environment and play equipment, implementation of policies and design of curriculum which had been shown to facilitate increased physical activity levels in children [18,19]. There is growing evidence for the benefits associated with physical activity programs that are implemented by teachers in pre-primary school settings (for example, see [20,21]). Yet, to the best of our knowledge, evidence supporting these approaches by teachers to promote physical activity in playgrounds or classrooms has yet to be synthesized.

It is of value to both teachers and researchers that the evidence is scoped on the effective strategies that enhance the physical activity levels of young children in early childhood education contexts. Published reviews have summarized the evidence of physical activity interventions mostly with a primary focus on obesity prevention [22,23,24,25] or on general health promotion [26,27,28,29,30]. A number of reviews have focused on the direct effects of physical activity promotion programs in children and adolescents [27,28,29,31] but only a few have solely focused on pre-primary school-aged children [18,19,32]. Those that are focused on early childhood have reviewed studies that were concerned about policy or environmental changes or were focused on outcomes other than physical activity (e.g., bone health, obesity prevention, motor skill development, etc.). A synthesis of evidence is needed on strategies or programs that teachers implement and deliver in school- or ECEC center-based settings to promote physical activity in young children and this current review aims to address this need. A scoping review is deemed appropriate, as literature spanning a broad range of study designs needs to be integrated given the relative paucity of consistently rigorous evidence [33]. A preliminary search of MEDLINE, the Cochrane Database of Systematic Reviews and the Joanna Briggs Institute (JBI) Evidence Synthesis was conducted, revealing no current or underway systematic reviews or scoping reviews on this particular topic.

### 1.2. The Present Review

To address the knowledge gaps of the existing literature, this scoping review aimed to identify strategies that are implemented by teachers to promote physical activity in kindergartens or ECEC services. The emphasis of this review is not on evaluating the quality of published studies which is typically in the realm of systematic reviews [34]. Instead, this review is focused on identifying available research in the area of physical activity promotion in early childhood education settings, with the view to generating an overview of the evidence that would support practitioners (e.g., teachers) or policymakers (e.g., school administrators) who might lack resources to find the evidence themselves [35]. The outcome of this scoping review is a synthesis of the evidence-based knowledge of physical activity promotion activities for early childhood and this would inform and help early childhood educators in providing opportunities for young children to have sustained physical activity engagement.

## 2. Methods

We followed the recognized framework proposed by Arksey and O’Malley [35] and the subsequent protocol suggested by Peters et al. [36] for carrying out and reporting scoping reviews (i.e., JBI framework). The protocol comprises four major stages: defining the scoping review question, developing the inclusion criteria, describing the approach of search strategy and summarizing and reporting the results. By adhering to this recommended framework, we gathered the evidence on the area of interest and did not attempt to critically appraise the methodology of reviewed articles [33,35].

### 2.1. Scoping Review Question

The research question was developed using the elements of Population, Concept and Context (PCC) following the adopted protocol [36]. The population of interest was pre-primary school-aged children (i.e., one to six years old), the concept was strategies or interventions that are implemented by teachers to promote physical activity and the context was school- or ECEC center-based settings. Early childhood is considered to encompass from birth to eight years of age but considering the age of entry in schools, children aged one to six years was selected as the population [37]. The following research question was established: “What strategies or interventions, that are implemented and delivered by teachers in school- or ECEC center-based settings, promote physical activity in pre-primary school-aged children?”

### 2.2. Identifying and Selecting Studies

The following six electronic databases were searched: PubMed, SCOPUS, PsycINFO, SPORT Discus, ERIC and Web of Science, to identify peer-reviewed literature. The Boolean operators “AND” and “OR” were utilized to strengthen the search strategy through multiple combinations. The following search phrase was constructed based on the PCC elements: (“Physical activity” OR “Physical education”) AND (“Early Childhood” OR “Preschool” OR “Kindergarten” OR “Child care”) AND (“Education” OR “Strategy” OR “Intervention”). We did not specify any terms related to “Teacher-led” or “Educator-led” at this stage since this might screen out any suitable articles that do not have such terms in the titles and/or abstracts. The identification of studies was performed in September 2020.

Studies were included if the reported interventions (1) were developed with a focus on promoting physical education/physical activity; (2) targeted early childhood (i.e., children aged one to six years); (3) were implemented in school/ECEC services; and (4) were implemented by center educators or teachers. Further, studies were included when (5) the reported primary outcome(s) included measurement(s) of physical activity levels; (6) they reported on original research and were published in peer-review journals; and (7) they were written in English.

**Figure 1:**
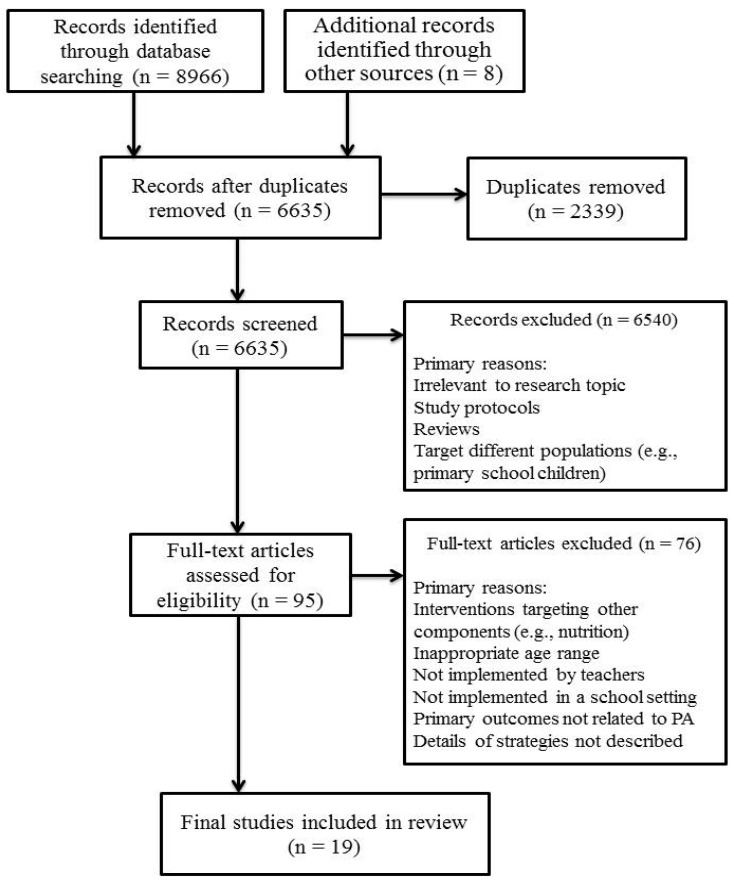
PRISMA flow diagram for article selection. PA stands for physical activity.

The electronic search strategy identified a total of 8966 articles and an additional eight articles were identified from other sources (i.e., from screening of reference lists). Duplicates from different databases were removed. A review of the abstracts discovered a large number of articles that were irrelevant to the research question and were therefore excluded, particularly those associated with school or state policies, observational or correlational studies that described physical activity levels and its correlation to other behavioral or psychological factors, interventions that focused primarily on obesity or healthy lifestyle and other populations (e.g., adolescents, primary school students, obese children, etc.). Any forms of protocols and reviews (e.g., systematic review, meta-analysis, etc.) were also removed. Ninety-five studies were identified as being relevant to the research question as guided by the inclusion criteria. Full-text versions of these articles were then obtained and independently examined by two reviewers. The process of article selection was based on the Preferred Reporting of Items for Systematic Reviews and Meta-Analyses (PRISMA) Statement [38] (see Figure 1) and reporting was guided by the PRISMA Extension for Scoping Reviews (PRISMA-ScR) [39].

### 2.3. Charting the Results

The themes and main issues of each study were identified by charting the data. Based on the protocol by Peters et al. [36], the following data were collected and charted: author(s), year of publication, research design, type of program, the aim of the study, study population and country of origin, duration of program, measures and outcomes, the existence of any process evaluation (an additional item for the purpose of this review) and detailed descriptions of the program. Data extraction was performed by one reviewer (T.C.T.M.) and the accuracy of extraction was verified by a second reviewer (C.M.C.).

## 3. Results

A total of 8974 records resulted from the initial search, including the eight additional articles identified from other sources. Upon removal of duplicates, 6635 potentially eligible articles remained. Of these 6635 articles, 6540 were excluded following examination of titles and abstracts, leaving 95 articles for full-text screening. After the removal of 76 full-text articles using the eligibility criteria, the final search output was 19 published articles that focused on strategies or programs that are implemented by teachers in school- or ECEC center-based settings to promote physical activity in children aged one to six years. Table S1 summarizes the study characteristics, types and details of programs and outcomes that were reported in the 19 studies.

### 3.1. Study Characteristics 

Thirteen out of the 19 studies were conducted in the United States, whereas three were from Australia and one each from Canada, Germany and Greece. There was none that came from any Asian territory (the largest and most populous continent). Most of the studies (68%) adopted the design of clustered randomized controlled trials (RCT), five of which randomized by class/classroom [20,40,41,42,43] and eight of which randomized by school/center [21,44,45,46,47,48,49,50]. The other five studies used a within-subject design [51,52,53,54,55] and the remaining one was classified as an observational study [56]. Nine of the 19 studies were conducted in preschools [20,21,40,41,42,43,45,50,51] and eight of them were conducted in ECEC services [46,47,48,49,52,53,54,55]. Aivazidis et al.’s [44] study was conducted in kindergartens whereas Dunn et al.’s [56] study was conducted in elementary schools (kindergarten sections). The interventions of all reviewed studies were implemented by school teachers [21,40,41,42,43,44,45,46,47,48,49,50,51,52,53,54,55,56], except Alhassan et al.’s [20] which was implemented by school teachers and research staff. Sample sizes across the included studies varied from five to 1154 participants. All of the 19 studies included both male and female participants.

### 3.2. Types and Details of Strategies

We identified ten types of strategies that were implemented by teachers: fundamental movement skills practice, musical activity, games, fitness training, coordination and perception training, behavioral skills training, integration with other curriculum areas, teacher participation (e.g., role modeling), mastery motivational climate and provision of equipment. Most of the included studies adopted more than one of the above strategies in their programs. Ten studies involved fundamental movement skills practice [40,41,43,44,46,47,48,49,53,55], eight of them implemented game elements [20,21,42,44,48,49,50,53], seven of them implemented musical activities [20,40,42,44,45,51,53], three of them provided portable equipment and supplies [21,46,48], three of them integrated physical activity with other literacies such as language and numeracy [21,42,52], three of them involved teacher modeling and/or participation in physical activity [21,46,51], three of them adopted mastery motivational climates [43,54,55], two of them involved fitness components [53,56], one of them incorporated physical activity-related behavioral skill training [41] and one of them emphasized coordination and perception skills [50].

The duration and frequency of the reviewed strategies widely varied. Program duration varied from three weeks [54] to about 11 months (one academic year) [50] in 17 of the 19 studies. The two remaining ones specified days of observation due to the nature of the study ([51]: 12 to 19 days of observation; [56]: one full school-day observation). In terms of frequency, eight of the 19 studies implemented physical activity sessions daily (or five days per week) [21,40,41,45,46,50,51,53], whereas five of them implemented the sessions three to four times per week [20,42,44,48,49] and three of them implemented them twice per week [43,54,55]. Duration of the implemented strategies ranged from a minimum of 10 min to a maximum of 60 min per session. Three studies did not specify the frequency of the strategies since they focused on training teachers to incorporate physical activity in their regular lesson plans [47,52,56].

### 3.3. Outcomes

The majority of articles evaluated the outcomes of physical activity levels by comparing a structured physical activity-related session delivered by teachers with a lesson guided by the usual daily school curriculum. A large number (68%) of articles used accelerometers to obtain the primary outcome of physical activity levels, in terms of minutes or percentage of time spent in sedentary, light or moderate and vigorous physical activity (MVPA) [20,21,40,41,42,43,45,47,48,49,50,53,55]. Some of them used pedometers to collect daily step counts [44,46] while the others used the Observational System for Recording Physical Activity in Children—Preschool version (OSRAC-P) [51,52] and direct observation [56]. One study used a heart rate monitor to measure heart rate and percentage of time spent above 50% resting heart rate which indicates vigorous physical play intensity [54]. Two studies used both accelerometers and OSRAC-P [42,45].

A large number (63%) of studies reported significantly higher physical activity levels for the intervention groups compared to the control groups, during the sessions and/or during school time, and/or in post-test or follow-up [20,21,41,42,43,44,47,48,52,54,55,56]. No significant changes in physical activity levels were observed in the intervention groups compared to the control groups in five of the 19 studies [40,46,49,50,53]. Brown et al.’s [51] study observed improvement in physical activity level in the intervention group based on comparisons of percentage intervals of MVPA but without statistical analyses. Alhassan et al.’s [45] study revealed significant but mixed results indicating increased time spent in light physical activity during intervention time but reduced school time spent in MVPA at the mid-point of intervention. The positive results of heightened physical activity levels observed in this review were mostly produced by strategies that involve fundamental movement skills practice (50%) and game elements (42%).

### 3.4. Process Evaluations

Five out of the 19 studies included process evaluations [21,42,45,48,49]. Wadsworth et al. [43,55] evaluated behavioral fidelity while Finch et al. [46] evaluated the implementation of the program and acceptability and reach. Dunn-Carver et al. [53] included ‘observer reports’ to assess whether the program was being implemented as intended.

Overall, a majority of these articles reported that the study interventions or programs have been largely implemented as intended and delivered successfully by teachers or center educators [42,43,46,48,49,53,55]. Only Alhassan et al.’s [45] study reported that teachers partially implemented the program as designed; 67.2% of teachers led the activities as instructed. Five studies evaluated participant’s responses and reported that children were generally enthusiastic and enjoyed participating in the programs [42,45,48,53]. Four studies reported teachers’ opinions and acceptability of the programs and three of them revealed that teachers or center educators were highly satisfied with the programs and resources [46,48,49]. The one other study reported that a majority of teachers stated the implementation of the program ‘took too long’ and such time constraints might explain why approximately one-third of their teachers did not implement the activities as planned [45].

## 4. Discussion

The purpose of this scoping review was to identify strategies or programs that are implemented by teachers to promote physical activity in early childhood education settings (i.e., kindergartens, preschools or ECEC services). The early childhood years are critically important for raising interest in physical activity and developing lifelong physical activity patterns [12,13,14,15]. As we recognize the important role of early childhood educators in creating opportunities and experiences for physical activities within the school setting [17], it is important to synthesize evidence of effective and in-context physical activity promotion strategies. Such synthesis would inform and help early childhood educators in designing curricula and learning activities that enable physical activity participation of young children.

**Figure 2:**
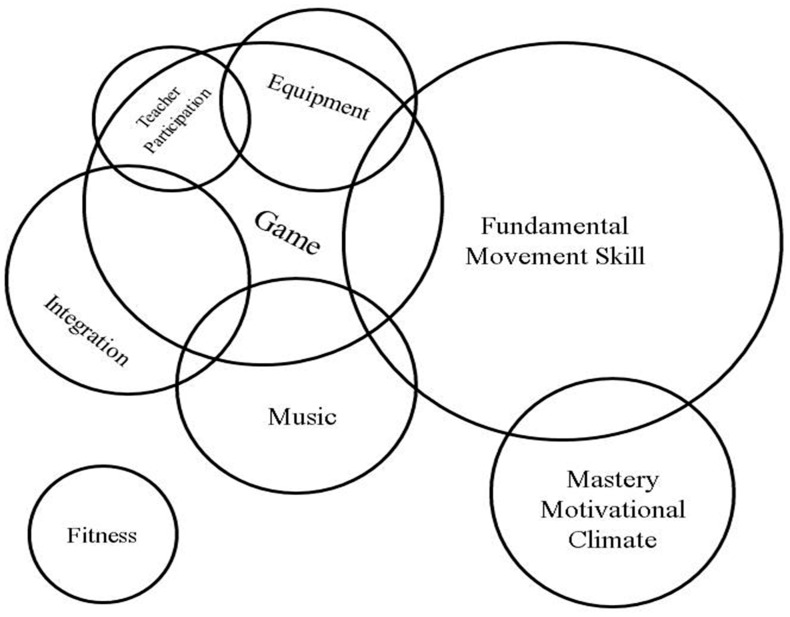
An overview of the types of successful strategies that were implemented by teachers or educators in school- or ECEC center-based settings. The area of the circle represents the quantity of studies associated with the strategy. The overlapping sector represents the existence (not quantity) of studies that incorporated the associated strategies.

The 19 published articles that were included in this review described various types of approaches that were implemented and delivered by educators with the intention of enhancing physical activity-related primary outcomes. We identified a total of ten types of strategies that were adopted among the reviewed studies, most of which implemented more than one type of the identified strategies in their programs. Figure 2 illustrates these strategies in terms of frequencies (i.e., larger circles represent more frequently reported strategies) and combinations (i.e., intersecting circles represent combinations). For instance, targeting fundamental movement skills was combined with a number of other strategies but targeting fitness was not combined with other strategies in the reviewed articles.

### 4.1. Types of Effective Strategies

The most commonly observed strategies in studies that reported effective programs were (1) fundamental movement skills practice and (2) game elements. Out of the 12 studies that revealed significant effects on enhancing physical activity levels [20,21,41,42,43,44,47,48,52,54,55,56], six of them focused on practicing fundamental movement skills [41,43,44,47,48,55]. Widely considered as the building blocks for more complex and specialized skills required for participation in a range of sport and recreational activities [57], fundamental movement skills comprise object control (e.g., throwing, catching), locomotor (e.g., jumping, leaping) and stability (e.g., turning, bending) skills that typically develop during childhood [58,59]. Apart from supporting physical and motor development, earlier systematic reviews have shown that fundamental movement skills are significant contributors to enhancing physical activity participation of young children [60,61]. Evidence includes those that are relevant for primary school-aged [62] and preschool-aged children [63]. Recent research also illustrates that the intensity of physical activity heightens during fundamental movement skills practice, especially when involving locomotor skills [64]. As such, findings from previously established evidence, combining with our current synthesis, collectively support a recommendation for early childhood education programs to include fundamental movement skills elements.

Apart from fundamental movement skills, five out of the 12 studies involved game elements [20,21,42,44,48]. In this review, studies that used game elements are those where teachers organized a series of games such as chasing after each other, dancing and playing games with balls, hoops, ropes and so forth. Other game elements included counting and math games (e.g., with balloons and scarves, etc.) and imagination games that related to concepts in other curriculum areas. The positive impact of implementing game elements in the programs is supported by existing literature, which argues that the choice of activity is mainly influenced by the level of enjoyment [65,66]. After all, one of the most often reported factors for participation in physical activity by children is ‘fun and enjoyment’ [67]. It is widely believed that positive and enjoyable experiences of physical activity in the early years will encourage children to continue enjoying and benefitting from physical activity as they grow into adulthood [68]. As such, our synthesis of evidence supports the use of game elements such that physical activities in early education settings are associated with high levels of enjoyment.

Fundamental movement skills practice and game elements might be the most commonly observed and effective strategies in our review but the strategies of integration and mastery motivational climate should not be neglected. All six studies that implemented either integration [21,42,52] or mastery motivational climate [43,54,55] have demonstrated significant positive effects on physical activity levels. Those that implemented integration are studies where physical activity opportunities were integrated into lesson plans of other curriculum aspects, including social studies and science, mathematics, language and arts. By integrating developmentally appropriate movement experiences into other learning areas within existing early childhood curricula, known implementation barriers related to stand-alone physical activity programs might be overcome [69] because additional time allocation might not be necessary in increasingly crowded curricula. Earlier evidence had shown enhanced learning outcomes in young children when integrating movement into daily learning experiences [70]. Although this type of approach has not been systematically evaluated in the existing literature, this could be a more viable and immediate solution to promote physical activity in schools and ECEC services. This might be especially true for societies where current priorities are largely focused on academic achievements and changes in perspectives would entail a longer-term and systemic cultural shift (e.g., East Asian societies such as Hong Kong [71]).

The reviewed studies that used the category of mastery motivational climate (also named task-involving climate) are those where children were encouraged more adaptive patterns of achievement behavior, including an intrinsic drive and motivation to master tasks, enhanced task persistence in the face of challenges and increased engagement in moderately challenging tasks without guidance from an adult [72,73]. There are six TARGET structures that must be implemented within an instructional setting by teachers to create a mastery motivational climate through which they deliver the curriculum and these structures have been successfully incorporated in education (see a review by [74]). The acronym “TARGET” represents task, authority, recognition, grouping, evaluation and time factors. A description of each TARGET structure is provided in Table S1 (i.e., Parish et al. [54] and Wadsworth et al. [55]). Through this climate, teachers encourage children to engage in a self-regulated learning process and develop self-referenced standards of success through their experiences. Researchers have investigated mastery motivational climates in physical education over the years. A considerable amount of work has demonstrated that mastery-oriented climates in physical education promote motor skill learning and physical activity more than teacher-directed (or performance-oriented) and free-play climates (see a review by [74]). Whilst the support for mastery motivational climates in early childhood physical activity promotion is not yet compelling, our current synthesis of evidence from this review suggests that the consistent evidence in physical education is likely to be applicable in early childhood education settings as well. There is reason to recommend that a mastery motivational climate may be considered by teachers to promote physical activity in early childhood education curricula.

It is important to mention that 8 out of the 12 studies with significant positive results in this review have implemented multiple strategies in their programs. While this review cannot conclude which combination(s) of strategies might produce the most effective outcomes on physical activity levels, the strategies mentioned in the above paragraphs could be used as a reference to guide practitioners (i.e., teachers and center educators) on which strategies to combine and incorporate into their curricula (see Figure 2 for better illustration). We note, nevertheless, that one of the more successful combinations observed in the current review is fundamental movement skills practice with a mastery motivational climate [43,55]. Stations of activities matching the fundamental movement skills can be seamlessly incorporated into the Task component of the TARGET structure while still closely following the other five components. Another successful combination observed is integration with games [21,42]. Considering the fact that game elements can be easily matched with any other strategies, this combination could provide extra benefits to children’s academic development. In particular, we note that the process evaluation by Trost et al. [42] revealed that children were enthusiastic, attentive and persistent in their learning activities of conventional learning areas (e.g., science, mathematics and language arts) when combined with game elements. However, the caveat is that the above combinations are recommendations based on the current review and we cannot rule out the possibility that other combinations could generate similarly positive (if not better) results despite the current lack of evidence.

We found inconclusive evidence for the duration and frequency of effective programs. Programs that succeeded in increasing physical activity levels delivered sessions with a duration ranged from one to nine months, two to five days per week and 10 to 50 min per session. Those that failed to increase physical activity levels delivered sessions with a duration ranged from 1.5 to 11 months, three to five days per week and 20 to 60 min per session. Interestingly, successful programs provided, on average, less frequent sessions (3.3 days/week and 30.3 min/session, versus 4.7 days/week and 36.7 min/session) over a relatively shorter period (4.3 months versus 5.8 months) compared to unsuccessful programs. Given the wide range of strategy types that were combined in the reviewed studies, this apparent trend in dosage (i.e., duration and frequency) effects may in fact be spurious. Rather than looking at dosage, we propose that the more critical factor is the degree of fit of programs with the local context (i.e., school environments and policies, teachers’ knowledge and availability, etc.). Furthermore, the quality of the strategies is perhaps the more important consideration for optimal outcomes, rather than the quantity of implementation sessions.

### 4.2. Common Challenges

While the current review highlights effective strategies that practitioners could pursue and deliver in their early childhood educational settings, it is essential to consider the common challenges and barriers that teachers might face when implementing the programs to promote physical activity. One of the barriers to incorporating physical activity in schools/ECEC services is the inclination for teachers or center educators to favor preparation work for formal schooling and other curriculum learning areas (such as numeracy and literacy) over physical activity opportunities [75]. This could be due to their insufficient understanding of the value and benefits that physical activity in early childhood can contribute to overall child development—including academic achievements [68]. Other recent findings also suggest that early childhood educators point to inadequate and insufficient resources related to physical activity that tend to negatively impact the quality of activities [76]. Lu and Montague [68] also raised concerns about teachers’ lack of adequate training and knowledge for developing and leading structured physical activity sessions in their classrooms. These might explain why less than 70% of teachers implemented and led the activities as planned in Alhassan et al.’s [45] study, which did not produce significant improvements in time spent in MVPA. Indeed, another reviewed article that failed to report significant benefits organized only a one-day training workshop for center educators, at which fewer than half of involved practitioners attended [46]. In addition, their follow-up support included only a two-hour site visit and two telephone contacts. In comparison, other effective programs have been characterized by up to five training sessions/workshops held on-site for teachers or center educators (for example, see [21]) and more frequent follow-up support (weekly on-site visits) (for example, see [42,44]). The need for training among early childhood educators and teachers for physical activity promotion has been documented recently, at least in places like Canada [77] and Hong Kong [78], where the higher education curricula for pre-service early childhood teachers have limited courses with a particular emphasis on physical activity, physical literacy or movement skill development. Considering the limited opportunities for learning and practicing the delivery of physical education in early childhood education settings, teachers have expressed the needs for more training opportunities and better access to resources associated with physical education [77].

Overall, our current review shows evidence that most programs that led to positive outcomes offered training sessions/workshops to teachers or center educators; three studies provided no such information [43,54,55]. As such, it is recommended that training opportunities coupled with continuous support should be made available to support pre-service and in-service teachers in integrating teaching strategies that promote physical activity in early childhood settings. Buckler and Bredin [77] proposed that training opportunities which are supported by ongoing education credits may facilitate the development of physical literacy for in-service early childhood educators. One of the reviewed articles [46] indicated that organizing training sessions on-site or providing several professional development opportunities scheduled at convenient times for teachers to attend and frequent follow-up support could increase the number of properly trained teachers to deliver programs. Professional development programs need to focus on ensuring that all teachers become capable of providing adequate quality physical activity opportunities for young children; age-appropriate knowledge about physical activity concepts and movement skill acquisition should also be enhanced [77]. As voiced by current educators, these opportunities should be interactive, experiential and meet teachers’ needs [76], that can take the form of educational seminars and workshops, and/or technology-enabled knowledge-sharing web portals. Web portals are becoming increasingly powerful tools to facilitate the discovery, acquisition and sharing of knowledge as they allow organizations and communities to work collaboratively, share ideas, publish documents and integrate scholarly information in easily accessible repositories [79].

### 4.3. Limitations

We acknowledge several limitations in this review. First, it should be noted that there are likely to be programs beyond the published literature (e.g., in the form of unpublished strategies used by schools or centers) which are not included in the present review. While unpublished programs may not allow us to systematically verify their benefits, we also cannot claim that this review encompasses all strategies that have been implemented. In addition, the fact that our review only included published studies could lead to publication bias; studies that report positive effects tend to be more frequently published than those that do not and studies that report null results tend to remain unpublished [80]. As such, there is a possibility that conclusions derived from our review might be limited and skewed by such bias. Second, most of the reviewed studies were conducted in the United States and none of them were from an Asian territory (the largest and most populous continent) nor from other developing regions such as those in Africa and South America. While the notable absence of literature from these other regions highlights the pressing need to strengthen physical activity promotion in early childhood education contexts globally, it also raises concerns about the generalizability of the current evidence to other regions and contexts. Practitioners (e.g., teachers) or policymakers (e.g., school administrators) in Asia, for example, need to carefully examine the design of the strategies, the observed outcomes and the potential feasibility and usefulness of the said strategies considering the diversity in school systems, infrastructure, culture and environments. Third, the heterogeneous methodologies of the reviewed studies presented a challenge to identifying best practice recommendations from the evidence. Based on this review, we are only able to suggest strategies that appear to be relatively more effective among others. Evidence is currently insufficient to support best practice standards that include information on program dosage and strengths of effects. Such conclusive recommendations would probably be possible when a sufficient volume of comparable research could be subject to a quality appraisal.

## 5. Conclusions

This review was designed to identify strategies or programs, implemented by teachers, which can improve physical activity levels in young children within kindergartens, preschools or ECEC services. The reviewed articles demonstrated various types of strategies associated with physical activity-related primary outcomes. Based on the published evidence to date, physical activity promotion in school- or center-based settings that are implemented by teachers is recommended to take a multi-strategy approach (e.g., the combination of fundamental movement skills practice and mastery motivational climate; integrating a game component in other learning areas), in conjunction with professional development training opportunities and continuous follow-up support for teachers. Future work is needed to fill the evidence gap in other regions (e.g., Asia, South America and Africa) that lack published literature related to the current topic. Finally, further research is needed to strengthen the evidence base (e.g., more rigorous study designs, adequate sample size, delayed follow-up of outcomes) to enable more robust best practice standards.

## Supplementary Materials

The following are available online at https://www.mdpi.com/1660-4601/18/3/867/s1, Table S1. Summary of characteristics, outcomes and details of programs of the included studies.
