# Supplementary material for: Strategies for Teachers to Promote Physical Activity in Early Childhood Education Settings—A Scoping Review
Source: Int J Environ Res Public Health. 2021 Jan 20;18(3):867. doi: 10.3390/ijerph18030867 (PMC7908495; doi:10.3390/ijerph18030867)
Supplement: Supplementary file 1 [file ijerph-18-00867-s001.pdf]

Table S1. Summary of characteristics, outcomes, and details of programs of the included studies.

Study	Design	Type	Aim	Target population & participants	Duration	PA measures and outcomes	Process Evaluation	Program description
Aivazidis et al. 2019	RCT; randomized by kindergartens	FMS; Music; Game	Evaluate the effect of a PA program ("Walk") led by teachers, on motor competence and PA of 5- to 6-year-old children	Greece kindergartens; 5-6 years old<br><br>n = 143;<br><br>mean age (yr) = 5.13 +/- 0.15	8 months	Step counts/day (pedometers)<br><br>Increased daily PA in post-test	no	Program components:<br><br>A) organized PE lessons (by PE teachers); 45-50mins/day, 4 days/week; gross motor activities, games, and music/movement activities (aiming at FMS development); B) PA during recess (by classroom teachers); chases, games with balls, hoops, ropes, etc.; C) walking opportunities (by classroom teachers); once a week; e.g., walking to the neighborhood supermarket/grocery/bakery; D) lectures, seminars, and training sessions for teachers (evaluation/follow-up every week)<br><br>Control: usual daily school curriculum
Alhassan et al. 2012	Pilot RCT; randomized by classrooms	FMS; Music	Examine the effects of a teacher-taught, locomotor skill-based PA program on locomotor skill and PA levels of minority	US preschools; low SES Latino/Hispanic and African-American children;	6 months	% of time spent in sedentary, light, and MVPA (accelerometers)<br><br>No significant effect on PA	no	Program components:<br><br>Designed to teach locomotor skill and movement concepts; 30mins/day, 5 days/week; 30 individual lesson plans; a typical lesson plan consisted of a 3–5 min low-intensity musical activity, 20 min of activities designed to teach one

			preschool-aged children	2.9-5 years old<br><br>n = 71;<br><br>mean age (yr) = 4.3 +/- 0.6				locomotor skill, and 5 min of extension activities designed to reinforce locomotor skill; training sessions for teachers (8hrs) without follow-up; have lesson plans with preparation sheets to aid teachers<br><br>Control : unstructured but supervised free playtime
Alhassan et al. 2013	Pilot RCT; randomized by classrooms	Music; Game	Explore the effects of increasing structured outdoor playtime on preschoolers' PA levels	US preschools; 2.9-5 years old<br><br>n = 67;<br><br>mean age (yr) = 4.1 +/- 0.8	4 weeks	% of time spent in sedentary, light, and MVPA (accelerometers)<br><br>Increased session PA, total daily and school-day PA (during the last week of intervention)	no	Program components (based on Sports, Play, and Active Recreation for Kids (SPARK) program):<br><br>Additional 30mins of outdoor playtime for 3 days/week; 12 lesson plans; each lesson plan consisted of a low-intensity musical activity of 3-5mins, an MVPA activity/game lasting 20-25mins, and a low/moderate-intensity musical activity of 3-5mins; training sessions for teachers (total 8hrs in 2 sessions) without follow-up<br><br>Control: any form of play in their usual in-school playground
Alhassan et al. 2016	RCT; randomized by centers	Music	Examine the effects of short bouts of structured PA implemented within the classroom setting as part of designated	US preschools; 2.9-5 years old<br><br>n = 291;<br><br>mean age (yr) = 4.1 +/- 0.8	6 months	% of time spent in sedentary, light, and MVPA (accelerometers); % intervals spent in sedentary, light, and MVPA (OSRAC-P)<br><br>Increased session PA across the	yes	Program components:<br><br>10-minute age-appropriate PA routines designed to elicit MVPA by engaging major muscle groups in the upper and lower body; 2x30mins/day, 5 days/week; implement the PA routines (set to music with simple movements that engage major muscle groups, available in DVD) during the first

			gross-motor playtime on preschoolers PA			intervention; decreased school-day PA at 3 months		10mins of their usual 30-min gross-motor playtime in their classroom; for the remaining 20 mins, preschoolers engaged in unstructured gross-motor playtime outside; have training sessions and refresher-training sessions for teachers<br><br>Control: unstructured gross-motor playtime
Annesi et al. 2013	RCT; randomized by classes	FMS; Behavioral skill	Evaluate the effects of a PA treatment (Start For Life) on PA and BMI in preschools	US preschools;<br><br>n = 1154;<br><br>mean age (yr) = 4.4 +/- 0.5	9 months	% of time spent in sedentary, vigorous, and MVPA (accelerometers)<br><br>Increased school-day PA (averaged across intervention)	no	Program components (Start For Life):<br><br>Emphasized building children's perceptions of mastery and ability through self-management/self-regulatory skills; 30mins/day, 5 days/week; incorporated primarily gross motor skills (e.g., walking, running, jumping) with behavioral skill training interspersed; PA patterns consisted of brief durations of high-energy PA followed by short rests; behavioral skills included long- and short-term goal-setting, self-monitoring of incremental progress, and acknowledgment of physical achievements; self-regulation and stress management techniques were also incorporated; training sessions for teachers (4 hrs); have daily lesson plans with preparation sheets to aid teachers<br><br>Control: 30 min of usual care
Brown et al.	Within-	Music;	Develop, implement,	US preschools;	range from	% intervals spent in MVPA	no	Program components (“Track Team” and “Dance Party”)

2009	subject design; single-case withdrawal (Study 1) and single-case alternating-treatment (Study 2)	Teacher participation	and validate acceptable and feasible teacher-implemented interventions to enhance young children's MVPA on playgrounds	n = 5 (Study 1 → n=3; Study 2 → n=2, at risk of school failure, and have frequent sedentary behavior and infrequent MVPA on the playground)<br><br>all age (yr) = 4	12 to 19 observations (or days)	(OSRAC-P)<br><br>Increased school-day PA (without statistical analyses)		(details of the teacher-implemented activities in the article) :<br><br>2x30mins/day; 1) Teacher-guided discussion of PA; 2) "Plan, Do, and Review" process for children's PA; 3) Teacher pep talk about participation after 5min on the playground; 4) Teacher participation in PA; 5) Teacher encouragement of PA; 6) Teacher acknowledgment of PA<br><br>Control: normal recess
De Marco et al. 2015	Within-subject design; single-case study, multiple baseline	Integration	Evaluate Be Active Kids, a program of PA developed for use with children from birth to 5 years old in their child care settings	US childcare centers; 1-5 years old<br><br>n = 116;<br><br>no mean age reported	Baseline: 5-11 weeks;<br><br>Intervention: 6-12 weeks	% intervals spent in sedentary, light, and MVPA (OSRAC-P)<br><br>Increased PA during intervention phase	no	Program components (based on Be Active Kids PA program):<br><br>no description for duration of session/day or /week; up to 40 activities for each age group and inserted into notebooks along with directions for setting up and leading each activity, adaptations to assist with the inclusion of children with disabilities, suggestions for ways to simplify or make the activity more challenging, and ideas for including content related to early numeracy and literacy; training sessions for teachers (2 hrs); provided notebooks and materials to aid teacher<br><br>Control: usual daily school curriculum

Dunn et al. 2012<br>*(extracted kindergarten grade)*	Observational study	Fitness	Evaluate the effects of Move-To-Improve (MTI), a classroom-based physical education program designed for kindergarten to third-grade teachers	US elementary schools;<br>n = 33 classrooms	1 full school-day observation	Non-standardized direct observation (including all instances of PA, how long those activities lasted and the category of the activity)<br>Increased school-day PA	no	Program components (based on Move-To-Improve (MTI) program):<br>One 3-hr MTI workshop: trainer discussed childhood obesity and the importance of PA, reviewed the MTI manual, led teachers through 17 of the 30 MTI fitness breaks, and asked teachers to discuss how they could adapt lessons to their classrooms; teachers received 1) a manual detailing 30 fitness breaks; 2) equipment kits including polyvinyl spot markers used to help children identify personal space, bean bags, scarves, and 2 CDs; and 3) a professional development stipend<br>Control: teachers were not trained in the MTI
Dunn-Carver et al. 2013	Within-subject design; pre-test/post-test (analyzed separately per center)	FMS; Music; Game; Fitness	Increase PA in preschool children enrolled in childcare centers by training childcare providers to deliver a PA curriculum	US child care centers; 4-5 years old<br>n = 32;<br>mean age (yr) = 4.3 +/- 1.5	6 weeks	MET/min or % of time spent in sedentary, light, and MVPA (SenseWear®Pro 3 armband); direct observation (PA activity categories)<br>No significant effect on PA	Observer reports: observe whether activities were being implemented as intended	Program components (based on Coordinated Approach to Child Health (CATCH) Early Childhood Education Curriculum (CEC)):<br>One-day teacher training: to implement at least 2 curriculum activity sessions/day (~60 mins/day) focused on the CEC PA components enhanced with play equipment, and stimulated by music and group games; I. Warm-up (3-5mins); II. Go Fitness (promote muscular strength, muscular endurance, and cardiovascular endurance); III. Go Activity (develop FMS and rhythm); IV. Limber Limbs (purposeful movement to

								improve muscular range of motion); V. Cool Down (cool down students' bodies and help with transition back to the classroom)
Finch et al. 2014	RCT; randomized by day care services	FMS; Teacher participation; Equipment	Evaluate the impact of a multi-level intervention on the PA levels of 3–5 year old children attending center-based childcare services	Australia day care services; 3-5 years old<br><br>n = 459;<br><br>no mean age reported	4 months	Step counts/min (pedometers)<br><br>No significant effect on PA	Implementation of intervention & intervention acceptability and reach	Program components:<br><br>1) daily structured FMS development sessions (20min, warm-up, teacher-led FMS, cool-down); 2) initiate PA structured teacher-led activities, incorporate active movements); 3) staff role modelling during child-initiated free play and delivery of instructional practices (provide verbal guidance and encouragement); 4) limiting children's small screen recreation and sedentary time (e.g., less than 30min at a time); 5) providing children with a PA promoting indoor and outdoor physical environment (existing resources and portable equipment more readily available); 6) training workshops for teachers (1 day); provide resources and instructional materials, deliver follow-up support, performance feedback and incentive<br><br>Control: did not receive the intervention
Goldfield et	RCT; randomized	FMS	Evaluate the efficacy of intervening with	Canada child care centers; 3-5 years	6 months	Intensities of PA, i.e., total, sedentary, light, MVPA,	no	Program components:

al. 2016	by centers		child care providers to increase overall PA levels and reduce adiposity in children aged 3–5 years attending licensed child care centers	old<br><br>n = 83;<br><br>Control (n = 43); mean age (yr)= 3.3 +/- 0.6;<br>Intervention (n = 40); mean age (yr) = 3.3 +/- 0.6		min/preschool day (accelerometers)<br><br>Increased school-day PA in post-test		Train-the-trainer approach; two 3-hr training workshops + resource training manual (Healthy Opportunities for Preschoolers): facilitate structured and unstructured PA that targeted locomotor skills, FMS, and gross motor skills through active play; included 12, 1-hr, biweekly “booster” sessions in the child care centers by the master trainer<br><br>Control: implemented their standard child care curriculum
Jones et al. 2011	Pilot RCT; randomized by centers	FMS; Game; Equipment	Assess the feasibility, acceptability and potential efficacy of a PA program for preschool children	Australia childcare centers; 3-5 years old<br><br>n = 97;<br><br>mean age (yr) = 4.13	20 weeks	Counts/min & % of time spent in sedentary, light, moderate, vigorous and MVPA (accelerometers)<br><br>Increased school-day PA (in the final 2 weeks of the intervention)	yes	Program components (‘Jump Start’):<br><br>A) professional development for staff/teacher; 4×30min workshops (include theory and practical components); B) structured lessons and unstructured activities for children; structured lessons (mainly teacher-facilitated), 3 times/week; each 20-min structured lesson focuses on 1 FMS with a number of components (through a series of fun activities and games); unstructured activities (mainly teacher-facilitated), offering an additional opportunity for the children to practice the skills learnt in the structured lessons (with specific equipment)<br><br>Control: continued their usual program

Jones et al. 2016	RCT; randomized by centers	FMS; Game	Evaluate a gross motor skill and PA program for preschool children which was facilitated solely by childcare educators	Australia early childhood centers; 3-5 years old<br><br>n = 150;<br><br>Control (n = 73); mean age (yr) = 4.0 +/- 0.6;<br>Intervention (n = 77); mean age (yr) = 4.0 +/- 0.6	6 months	% of time spent in sedentary, light, MVPA and LMVPA (accelerometers)<br><br>No significant effect on PA	yes	Program components ('Jump Start'):<br><br>A) professional development for staff/teacher; 4×30min workshops (include theory and practical components); B) structured lessons and unstructured activities for children; structured lessons (mainly teacher-facilitated), 3 times/week; each 20-min structured lesson focuses on 1 gross motor skill with a number of components (through a series of fun activities and games); unstructured activities (mainly teacher-facilitated), offering an additional opportunity for the children to practice the skills learnt in the structured lessons (with specific equipment)<br><br>Control: continued their usual program
Parish et al. 2007	Within-subject design	Mastery Motivational Climate	Determine the effectiveness of a planned mastery motivational physical play session on physical activity in toddlers as compared to a non-planned free play session	US day care centers; 1.5-3.2 years old (18-38 months)<br><br>n = 21;<br><br>mean age (months) = 31 +/- 5.5	3 weeks	Mean heart rate (HR) (Actiheart Monitor); % of time spent above 50% resting HR → vigorous physical play intensity (Physical Activity Heart rate-50 (PAHR>50))<br><br>Increased session PA (averaged across intervention)	no	Program components:<br><br>30 mins/session, 2 times/week; 6 dimensional TARGET structure: Task: 12 meaningful and developmentally appropriate motor activities with multiple skill difficulty; Authority: children choose physical play activities in which they preferred to participate; Recognition: teachers privately recognize children based on their level of effort and engagement in mastery behaviors, with verbal and visual corrective feedback and encouragement; Grouping: children decide with whom they wanted to play; Evaluation: children

								evaluate themselves, and teachers provided evaluative feedback (privately and encouraging); Time: children explore an activity on their own (have optimum time to practice and learn to proficiency); teachers gave verbal cues to help managing the time<br><br>Control: free play condition
Pate et al. 2016	RCT; randomized by preschools	Game; Integration; Teacher participation; Equipment	Test the effects of an adaptable ecologic preschool intervention, implemented by preschool teachers, on the PA of young children	US preschools; 3-5 years old<br><br>n = 379;<br><br>Control (n = 191); mean age (yr) = 4.5 +/- 0.4;<br>Intervention (n = 188); mean age (yr) = 4.5 +/- 0.4	From fall to spring (15-31 weeks)	Minutes/hour of total, sedentary, light and MVPA in preschool (accelerometer)<br><br>Increased school-day PA during follow-up	yes<br><br>(http://dx.doi.org/10.1186/1471-2458-13-728)	Program components (The Study of Health and Activity in Preschool Environments (SHAPES)):<br><br>1) structured, teacher-led PA opportunities in the classroom (e.g., dancing, obstacle courses) (at least 10mins/day); 2) structured and unstructured PA opportunities at recess (e.g., races, follow the leader) (at least 20 mins/day); 3) PA integrated into pre-academic lessons; 4) modifications were made to the preschool social and physical environments: teacher verbal encouragement and participation in PA, and activities that involve MVPA (e.g., dancing, chase games, ball games); providing PA supplies (e.g., balls, music, scarves); 5) initial teacher training 2-3 hrs; 5 teacher workshops through the intervention; site-visit (2 visits/month during 2nd yr)<br><br>Control: continued their regular practice

Roth et al. 2015	RCT; randomized by preschools	Game; Coordination and perception	Evaluate a multi-component, child-appropriate preschool intervention program led by preschool teachers to enhance PA and motor skill performance (MS) in 4- and 5-year old children	Germany preschools; 3-6 years old<br><br>n = 709;<br><br>mean age (yr) = 4.7 +/- 0.6	about 11 months (1 academic year)	Change in % of (wearing) time spent in MVPA (accelerometer)<br><br>No significant effect on PA	no	Program components (The Prevention through Activity in Kindergarten Trial (PAKT)):<br><br>Daily 30-min PA lesson (receive PA homework once or twice/week); main focus: enhance coordinative skills and perception (optical, acoustical, tactile, vestibular, kinesthetic); the standardized structure: an initial ritual → an introduction period where teachers encourage children in developing their motor skills and attending to joyful games and exercise tasks → a cool-down → feedback; 2 training workshops for teachers; provide a collection of games and exercise tasks for the daily PA lessons, and a manual including pedagogical, didactical and methodological background information<br><br>Control: continued their routine schedule
Trost et al. 2008	RCT; randomized by classrooms	Game; Music; Integration	Evaluate the effect of a “move and learn” curriculum on PA in 3- to 5-year-olds attending a half-day preschool program	US preschools; 3-5 years old<br><br>n = 42;<br><br>Control (n = 22); mean age (yr) = 4.0 +/- 0.7<br><br>Intervention (n =	8 weeks	Minutes of VPA and MVPA (accelerometer); % MVPA during circle time, outdoor and indoor free choice, snack time, and transitions (OSRAC-P)<br><br>Increased school-day PA (mainly over the final weeks of intervention)	yes	Program components (‘move and learn’ curriculum):<br><br>Opportunities for PA were integrated into all aspects of the preschool curriculum, i.e., math, social studies and science, language arts, and nutrition education; activities included counting and number-recognition games using scarves and balloons and music-based chasing and imagination games addressing concepts in language arts, science, social studies, and nutrition education (e.g., letter launch, musical march, animal antics, broccoli broccoli, and pizza delivery man);

				20); mean age (yr) = 4.1 +/- 0.7				include (at least) 2 ‘move and learn’ curriculum activities lasting 10 mins or longer in each 2.5-hour session; session offered 4 days/week; single 3-hr training session for teachers; provide video demonstrating the different activities; weekly follow-up<br><br>Control: completed their usual early childhood curriculum
Wadsworth et al. 2017	Within-subject design	FMS; Mastery Motivational Climate	Determine and compare children’s participation in PA during a mastery-motivational climate changed during a 20-week intervention to children’s free-play activity during a typical day at their local day-care facility	US day care centers;<br><br>n = 12;<br><br>all 4 years old	20 weeks	% of time spent in sedentary, light and MVPA (accelerometer)<br><br>Increased session PA (accumulated across intervention)	Behavioral fidelity	Program components:<br><br>45mins/session, 2 times/week; 6-8 stations for practice of various fundamental motor skills (i.e., locomotor and object control skills); 6 dimensional TARGET structure: Task: 12 meaningful and developmentally appropriate motor activities with multiple skill difficulty; Authority: children choose physical play activities in which they preferred to participate; Recognition: teachers privately recognize children based on their level of effort and engagement in mastery behaviors, with verbal and visual corrective feedback and encouragement; Grouping: children decide with whom they wanted to play; Evaluation: children evaluate themselves, and teachers provided evaluative feedback (privately and encouraging); Time: children explore an activity on their own (have optimum time to practice and learn to proficiency); teachers gave verbal cues to help managing the time

								Control: unstructured free play condition
Wadsworth et al. 2020	RCT; randomized by classrooms	FMS; Mastery Motivationa l Climate	Determine which type of intervention, PA or FMS focus, promoted greater levels of participation in PA for all preschool-aged children during outdoor play	US preschools; 3-5 years old<br><br>n = 98;<br><br>mean age (yr) = 4.48	7 weeks	% of time spent in MVPA (accelerometer)<br><br>Increased session PA (in the final 2 weeks of intervention) (only in males)	Behavioral fidelity	Program components:<br><br>Consisted of 6-8 stations that focused on FMS (hopping, running, galloping, jumping, throwing, catching, striking and dribbling) and physical activity; using a high autonomy climate (children free to choose) (i.e., mastery motivational climate); 30mins/session, 2 times/week. FMS: encouraged the children to learn motor skills by providing constant instruction and feedback regarding the technique and form of the skills at each station; PA: encouraged the children to participate in as much PA as possible at each station (never gave instruction on skill development); FMS+PA: equally encouraged the children to perform their skills correctly and get as much PA as possible<br><br>Control: unstructured free play

Note: PA = physical activity; RCT = randomized controlled trial; FMS = fundamental movement skills; MVPA = moderate to vigorous physical activity; US = the United States; SES = socioeconomic status; OSRAC-P = Observational System for Recording Physical Activity in Children - Preschool version
